# Adaptation Evolution and Phylogenetic Analyses of Species in Chinese *Allium* Section *Pallasia* and Related Species Based on Complete Chloroplast Genome Sequences

**DOI:** 10.1155/2020/8542797

**Published:** 2020-06-11

**Authors:** Fu-Min Xie, Deng-Feng Xie, Chuan Xie, Yan Yu, Song-Dong Zhou, Xing-Jin He

**Affiliations:** Key Laboratory of Bio-Resources and Eco-Environment of Ministry of Education, College of Life Sciences, Sichuan University, Chengdu 610065, China

## Abstract

The section *Pallasia* is one of the components of the genus *Allium* subgenus *Allium* (Amaryllidaceae), and species relationship in this section is still not resolved very well, which hinders further evolutionary and adaptive studies. Here, the complete chloroplast genomes of five sect. *Pallasia* species were reported, and a comparative analysis was performed with other three related *Allium* species. The genome size of the eight species ranged from 151,672 bp to 153,339 bp in length, GC content changed from 36.7% to 36.8%, and 130 genes (except *Allium pallasii*), 37 tRNA, and 8 rRNA were identified in each genome. By analyzing the IR/LSC and IR/SSC boundary, *A. pallasii* exhibited differences compared with other seven species. Phylogenetic analysis achieved high supports in each branch, seven of the eight *Allium* species cluster into a group, and *A. pallasii* exhibit a close relationship with *A. obliquum*. Higher pairwise Ka/Ks ratios were found in *A. schoenoprasoides* compared to *A. caeruleum* and *A. macrostemon* while a lower value of Ka/Ks ratios was detected between *A. caeruleum* and *A. macrostemon*. This study will be a great contribution to the future phylogenetic and adaptive research in *Allium*.

## 1. Introduction

Genus *Allium* L. is one of the largest monocotyledonous genera, including about 950 species [1], and it is the only genus of Allioideae in the new APG IV [2]. This genus has a major center from the Mediterranean Basin to Central Asia and Pakistan and a second less pronounced one located in the western North America [3, 4]. Many important economical species, like onion, shallot, and scallion, were included in this genus, and some of them are regarded as vegetables or spices, even as ornamental plants like *A. caeruleum* [5]. Based on the phylogenetic study of ribosomal DNA-ITS sequences, the genus has been classified into 15 subgenera and 72 sections [6]. This research advanced that this genus *Allium* is a monophyletic group and evolved proceeded in three major clades. Species relationships in the first and second clades were almost clear, but those in the third clade were complex; in particular, species relationships among subgenera *Rhizirideum*, *Allium*, *Cepa*, *Polyprason*, and *Reticulatobulbosa* were still not well resolved [4, 7].

The section *Pallasia* is the largest section in the genus *Allium* subgenus *Allium* (Amaryllidaceae) in China. This section was firstly established by Friesen et al. with *A. pallasia* as the type species [6]. According to morphological study and molecular biological data, Li et al. divided the Chinese *Allium* species into 13 subgenera and 34 sections, and among them, *A. delicatulum*, *A. eusperma*, *A. pallasii*, *A. glomeratum*, *A. schoenoprasoides*, *A. songpanicum*, and *A. tanguticum* were classified into sect. *Pallasia* [4]. These species have a wide distribution and complex morphological diversity, in which *A. schoenoprasoides* grows in a grassy slope 3000 meters above the sea level, and *A. pallasii* grows in a dry slope with an altitude of 500 m [8]. Previous phylogenetic analysis showed that sect. *Pallasia* is not monophyletic, and parallel branches in phylogenetic trees made it difficult to understand the phylogenetic relationships of species in this section only using ITS and chloroplast gene fragment [4]. Polymorphism and homoplasious characters of these species also made it difficult to recognize phylogenetic position using the traditional classification method [4, 6, 7, 9].

The chloroplast is an important plastid, which provides necessary energy for growth by photosynthesis and plays vital roles in physiology and development of plant. It has a typical quadripartite circular DNA genome, mostly ranging from 120 to 160 kb, and includes a large single copy region (LSC), a small single copy region (SSC), and two inverted repeats (IRs) [10–12]. Cp genomes are haploid, and most of them are maternally inherited. High conservation of gene content and genome structure makes cp genome an important resource for reconstructing the phylogenetic relationships among plant groups [13–20], which may suggest that the cp genome could be better to solve the relationships between species [21–23]. Additionally, the cp genome encodes many chloroplast-specific components, like photosynthesis genes, which play fundamental roles in the life of plant [24–26]. A recent study showed that the adaptation of sunlight preferences was related to adaptation evolution of chloroplast genes [27, 28]. The differences of structural information in chloroplast genome may play an important role; previous studies suggested that the similarity of the IR region of some species reflected that they may have a common ancestor [29]. The comparative analysis of chloroplast genome can provide more information for phylogenetic research like highly divergent regions [28]. Other studies suggested that the selective pressure in chloroplast genomes has played a key role in *Allium* species adaptation and evolution [30, 31]. Although these studies have demonstrated that complete chloroplast genome has extraordinary advantages in phylogenetic analysis, the species in sect. *Pallasia* were not investigated, and the systematic relationships between these species are not clear until now.

In this study, we reported the complete chloroplast genomes of eight species (*A. delicatulum*, *A. pallasii*, *A. schoenoprasoides*, *A. songpanicum*, *A. tanguticum* from sect. *Pallasia*, and three related species *A. caeruleum*, *A. teretifolium*, and *A. macrostemon*) and performed comparative analyses. The objectives of this study were (1) to explore the overall structural pattern of the eight plastid genomes, (2) to solve the phylogenetic relationships between these species based on cp genome sequences, and (3) to analyze the adaptive evolution and selection pressures of *Allium* species based on chloroplast genomes.

## 2. Materials and Methods

### 2.1. DNA Extraction, Sequencing, and Annotation

The modified CTAB method [32] was used to extract genomic DNA from dried leaves of *A. delicatulum*, *A. pallasii*, *A. schoenoprasoides*, *A. songpanicum*, *A. tanguticum*, *A. caeruleum*, and *A. teretifolium.* Genomic data of these species were sequenced using an Illumina Hiseq 2500 platform by Biomarker Technologies, Inc. (Beijing, China). The collection information and Genebank accession numbers are in Table S1. Seven chloroplast sequences were completed with NOVOPlasty [33], a fast *de novo* assembler, and the seed sequence is the *rbcL* from the *Allium cepa* (KM088014); contig information is in Table S2. Contigs generated by NOVOPlasty were sorted and joined into a single draft sequence with *A. cepa* as the reference in the software Geneious [34]. Chloroplast genomes were annotated using PGA (Plastid Genome Annotator) [35] with two reference sequences *A. cepa* (KM088014) and *Amborella trichopoda* (AJ506156) which has the highest gene numbers among known gymnosperms and angiosperms; after that, sequences were adjusted using Geneious manually [34]. These plastid genome maps were generated using OGDRAW [36].

### 2.2. SSR Characterization

The MISA-web was used to find microsatellites (SSRs) in the eight *Allium* cp genomes [37]. The parameters are set to ten, five, four, three, three, and three for mononucleotide (mono-), dinucleotides (di-), trinucleotides (tri-), tetranucleotides (tetra-), pentanucleotide (penta-), and hexanucleotides (hexa-), respectively.

### 2.3. Phylogenetic Analyses

In order to clarify the phylogenetic relationship of species (*A. delicatulum*, *A. pallasii*, *A. schoenoprasoides*, *A. songpanicum*, *A. tanguticum*, *A. caeruleum*, *A. teretifolium*, and *A. macrostemon*), we download 22 cp genome sequences from Genebank, including 20 *Allium* species (Table S3), *Agapanthus coddii* (KX790363) from Agapanthoideae, and *Narcissus poeticus* (NC_039825) from Amaryllidoideae as the outgroups. All 30 cp genome sequences were used for phylogenetic analysis. Because molecular evolutionary rates were different in the whole cp genome, we built the phylogenetic trees based on the following three datasets: (1) complete chloroplast genomes, (2) the combined coding sequences, and (3) LSC regions. Firstly, all the sequences were aligned with MAFFT v. 7.1.2.9 [38] and adjusted with MEGA 7.0 [39] manually. Then, we used RAxML 8.2.8 [40] to perform the maximum likelihood (ML) analyses with the GTR+G model for 1000 replications. And Bayesian inference (BI) was performed in MrBayes v. 3.2.5 [41] under the GTR+G model. The Markov chain Monte Carlo (MCMC) algorithm was performed for 1,000,000 generations, trees were sampled every 1000 generations for each data partition, the first 20% of trees were discarded as burn-in, and the remaining trees were used to build a 50% majority-rule consensus tree.

### 2.4. Genome Comparative Analyses

The IR regions have been marked at the time of annotation using PGA [35]. IR/SSC and IR/LSC boundary analyses among the eight *Allium* species and the outgroups *Agapanthus coddii* and *Narcissus poeticus* were compared in Geneious [34].

Pairwise chloroplast genomic alignment among eight species was compared by mVISTA in the Shuffle-LAGAN mode [42], and *Allium cepa* (KM088014) was used as a reference.

The relative synonymous codon usage (RSCU) among the eight *Allium* species was calculated using DnaSP version 6 [43].

Combined CDS (coding sequences) of 42 photosynthesis genes were aligned using MAFFT v. 7.129 [38] for 30 cp genomes in phylogenetic analysis. Ka/Ks ratios were calculated using KaKs_Calculator 2.0 [44].

## 3. Results

### 3.1. Chloroplast Features of These Allium Species

**Figure 1:**
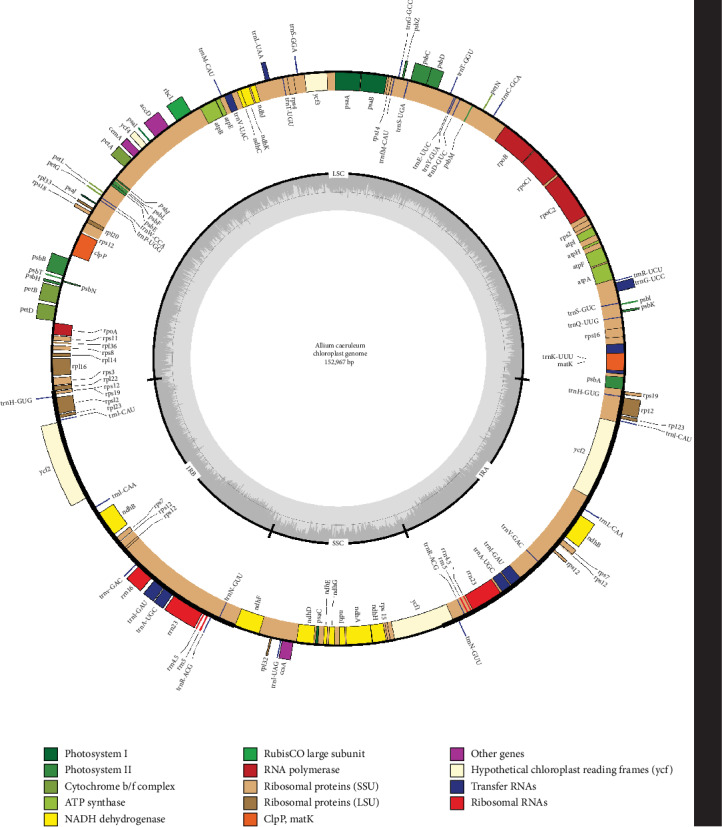
Gene map of *Allium* chloroplast genomes (represented by *A. caeruleum*). Genes outside the circle are transcribed clockwise, and genes shown on the inside of the circle are counterclockwise. Genes belonging to the functional group are color-coded. The darker gray in the inner corresponds to GC content, and the lighter gray corresponds to AT content. IR = inverted repeat; SSC = small single copy; LSC = large single copy.

The eight *Allium* complete cp genome sequences ranged from 151,672 bp (*A. pallasii*) to 153,339 bp (*A. teretifolium*) in length. All eight cp genomes showed a typical quadripartite structure (Figure 1), which consisted of a pair of IR regions (26,343–26,541 bp) separated by the LSC (80,924–82,539 bp) and SSC (17,600–18,064 bp) regions. The GC content is 36.7–36.8%, indicating nearly identical levels among the eight *Allium* cp genomes (Table 1). The total number of annotated genes varies between 128 and 130, including 83–85 protein-coding genes, eight rRNA genes, and 37 tRNA genes, respectively (Table 2). The length, GC content, and gene components of the eight species were included in Table 1.

### 3.2. SSR Analysis of These Species and Codon Usage

**Figure 2:**
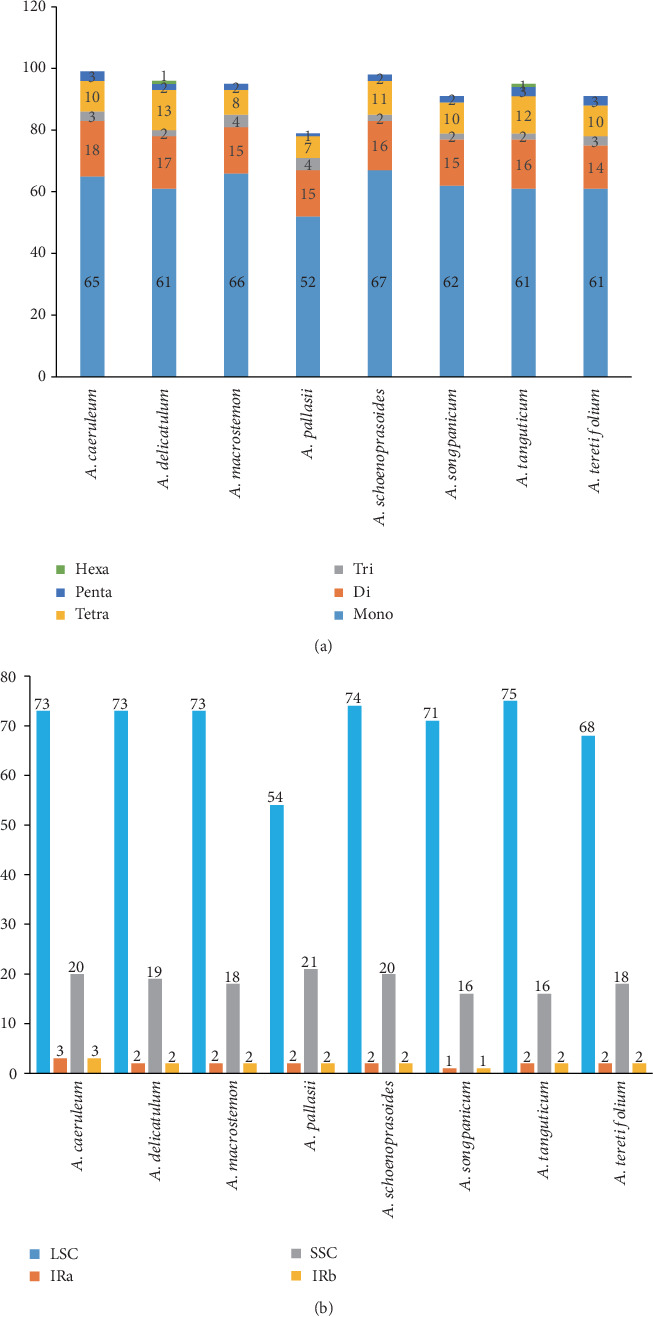
(a) Microsatellite loci in the eight chloroplast genomes for mono-, di-, tri-, tetra-, penta-, and hexanucleotides. (b) Number of SSRs in the LSC, IR, and SSC regions in eight *Allium* chloroplast genome sequences.

We used MISA-web to find microsatellites (SSRs) in the eight cp genomes, and six types of perfect SSRs were found. In total, 99, 96, 95, 79, 98, 91, 95, and 91 SSRs were detected in *A. delicatulum*, *A. macrostemon*, *A. pallasii*, *A. schoenoprasoides*, *A. songpanicum*, *A. tanguticum*, *A. caeruleum*, and *A. teretifolium*. Only *A. delicatulum* and *A. tanguticum* have hexanucleotide repeats, which is special in these species. The largest group of SSRs was mononucleotide repeats, ranging from 52 to 67 in these eight species, making up more than half of SSRs (Figure 2(a)). Most SSRs are located in the LSC region (Figure 2(b)), which is identical with previous research studies [28]. The relative synonymous codon usage (RSCU) was calculated in Table S4.

### 3.3. IR/SSC and IR/LSC Boundary

**Figure 3:**
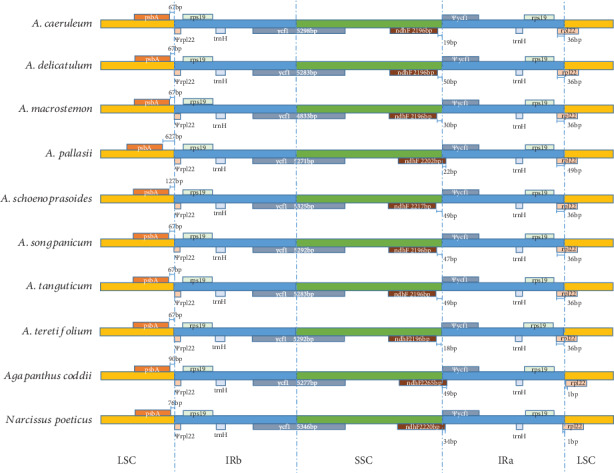
Comparison of LSC, SSC, and IR border regions among eight *Allium* cp genomes. Colored boxes for genes represent the gene position.

The expansion and contraction of the IR region in chloroplast are the main reason for the change of chloroplast genome size [45–47]. The expansion of the IRs to *rps19* or *rpl22* (Figure 3), which has been described by previous studies, was also found in our results. The *ndhF* gene flanked the junction between SSC and IRb in *A. pallasii*, *Agapanthus coddii*, and *Narcissus poeticus* while 22 bp–49 bp was located in the IRa region, but the *ndhF* gene clusters with a length of 18–50 bp away from the SSC/IRa boundary in the other seven *Allium* species. The *psbA* gene also has different positions in these species; the length from *psbA* gene to the LSC/IRb boundary in *A*. *pallasii* (627 bp) was much longer than that in other species (67–127 bp). There are also some differences in positions of *rpl22* gene, 36 bp-49 bp was located in the IRa region in eight *Allium* species, but *rpl22* gene was almost located in the LSC region in *Agapanthus coddii* and *Narcissus poeticus*.

### 3.4. Nucleotide Diversity and Sequence Identity Plot

**Figure 4:**
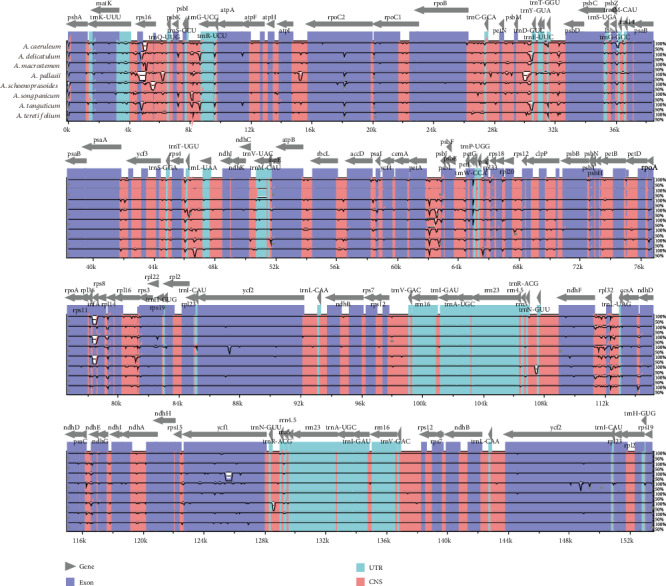
Visualization alignment of eight *Allium* cp genomes. VISTA-based identity plot showing sequence identity among eight *Allium* species using *A. cepa* as a reference.

The mVISTA software was used to compare the complete chloroplast genome of these eight species; the annotation of *A. cepa* (KM088014) was used as a reference. The alignment revealed a high sequence similarity across eight *Allium* plastid genomes, which showed that the genomes were highly conserved (Figure 4). Highly divergent regions among eight *Allium* chloroplast genomes were mainly located in the intergenic spacers, including *trnK-UUU*–*trnQ-UUG*, *trnS-GCU*–*trnR-UCU*, *rpoB*–*psbD*, *rps4*–*trnL-UAA*, *petA*–*rpl20*, and *ndhF*–*ndhD*, but some protein-coding regions also have distributions like *ycf1*.

### 3.5. Phylogenetic Analysis

**Figure 5:**
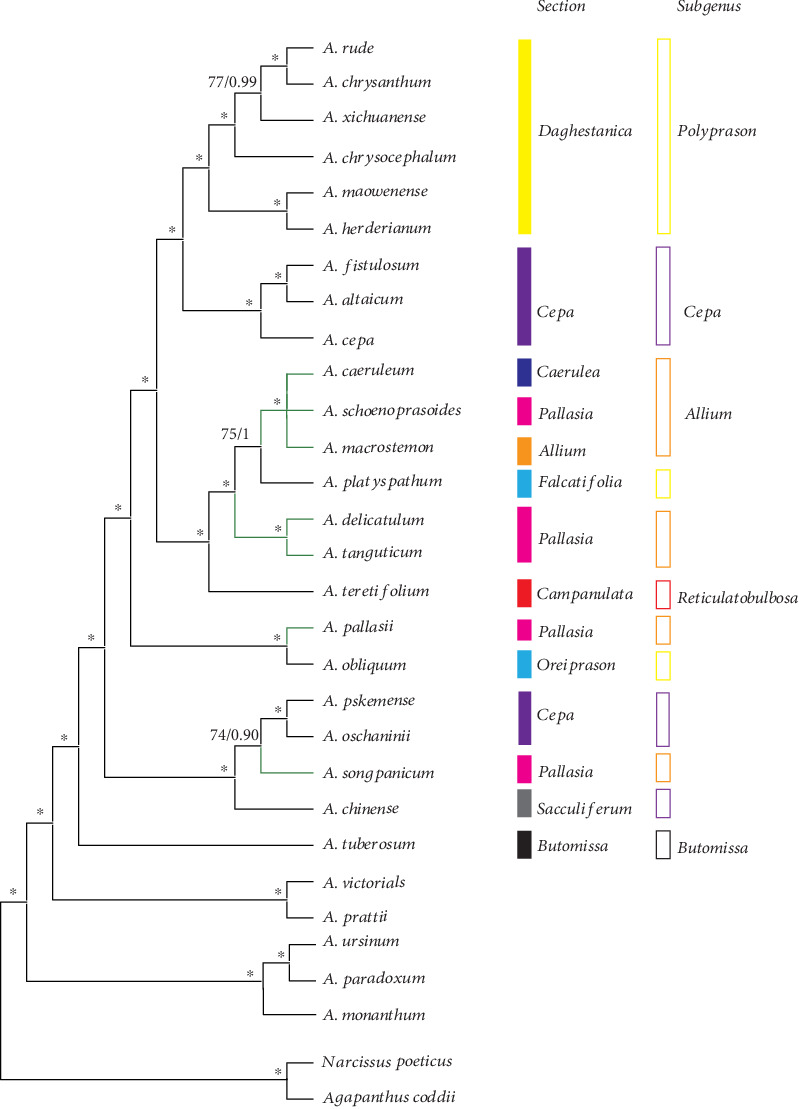
The phylogenetic relationships of these eight *Allium* species with other 22 related species based on whole genome sequences. Tree constructed by Bayesian inference (BI) and maximum likelihood (ML) with the posterior probabilities of BI and the bootstrap values of ML above the branches, respectively. ∗ represents maximum support in all two analyses.

The chloroplast genomes have showed great potential in reconstructing the phylogenetic relationships among plant groups [17, 48–51]. A phylogenetic tree of 28 *Allium* species and their outgroups (*Narcissus poeticus* and *Agapanthus coddii*) was constructed in this study. For Bayesian inference (BI) and maximum likelihood (ML) of three datasets, the posterior probabilities and bootstrap values were very high for each lineage, and only a clade had a relatively low posterior (Figure 5) (Figure S1). Both the maximum likelihood (ML) and BI phylogenetic results strongly supported that *A. macrostemon*, *A. caeruleum*, and *A. schoenoprasoides* were the closest species in the phylogenetic trees based on three datasets, which is the same as the previous study based on ITS sequences. These 3 species and *A. platyspathum* formed the sister groups to the clade of *A. delicatulum* and *A. tanguticum*. These 6 species and *A. teretifolium* proposed a sister relationship to the clade formed by the species from sect. *Daghestanica* (*A. chrysanthum*, *A. rude*, *A. xichuanense*, *A. chrysocephalum*, *A. maowenense*, and *A. herderianum*) and sect. *Cepa* (*A. fistulosum*, *A. altaicum*, and *A. cepa*). And *A. chinense*, *A. songpanicum*, *A. pskemense*, and *A. oschaninii* had a close relationship.

### 3.6. The Ka/Ks Ratios of Species Pairwise

**Figure 6:**
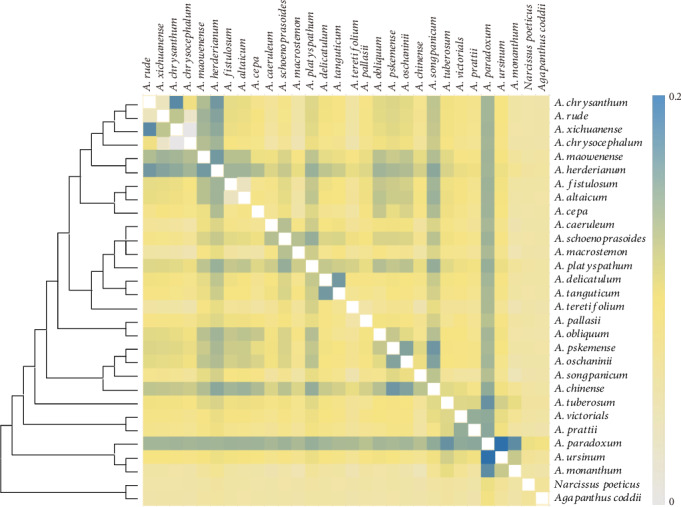
Pairwise Ka/Ks ratios in *Allium* (Allioideae) and their outgroups. This heat map shows pairwise Ka/Ks ratios between every sequence in the multigene nucleotide alignment.

The pairwise Ka/Ks ratios of photosynthesis gene single-CDS of each species pair were calculated (Figure 6). Higher pairwise Ka/Ks ratios were observed between *A. schoenoprasoides* and *A. macrostemon*, *A. schoenoprasoides*, and *A. caeruleum* rather than that between *A. macrostemon* and *A. caeruleum*. *A. delicatulum* and *A. tanguticum* also had distinctive Ka/Ks ratios. There were higher pairwise Ka/Ks ratios in the clade formed by *A. chinense*, *A. songpanicum*, *A. pskemense*, and *A. oschaninii*.

## 4. Discussion

### 4.1. The Intergenetic Analysis of Species in Sect. *Pallasia* and Related Species

In this study, a phylogenetic tree with high support values was obtained by analyzing 30 cp genomes from *Allium* species. Three species (*A. caeruleum*, *A. schoenoprasoides*, and *A. macrostemon*) show well support in the cp genome phylogenetic tree, which had been found in the previous studies [4, 52], these three species all have distinctive common-possessed bulbs and special fistulose leaves, and these might demonstrate their close relationships [8, 53].

*A. tanguticum* and *A. delicatulum* used to be positioned into sect. *Pallasia* [4]; however, our phylogenetic analysis results show that *A. tanguticum* and *A. delicatulum* do not exhibit close relationship with the type species of sect. *Pallasia* (*A. pallasii*); phylogenetic results strongly support that these two species are closely clustered with *A. platyspathum*, *A. caeruleum*, *A. schoenoprasoides*, and *A. macrostemon.* But our results show that *A. platyspathum* from subgenus *Polyprason* sect. *Falcatifolia* has a close affinity with species in subgenus *Allium*, which is not consistent with the classification based on morphology. *A. songpanicum* used to be positioned into sect. *Pallasia* based on morphology (Li et al., 2010); it has a similar bulb like *A. pallasii*. However, our results show that *A. songpanicum* is clustered with *A. chinense*, *A. pskemense*, and *A. oschaninii*, and they present a monophyletic clade. *A. pallasii* used to be an isolated clade, and it was the type species of the sect. *Pallasia* in subgenus *Allium*, but it shows close relationship with *A. obliquum* from sect. *Oreiprason* in subgenus *Polyprason* [4, 6, 9], and it has far relationships with species in subgenus *Allium*. Therefore, the phylogenetic inference of *A. pallasii* is still unclear; more *Allium* species in Central Asia is needed to solve the phylogenetic position of it.

Homoplasious characters of these species may make it difficult to recognize their relationship from morphological characteristics [4, 6, 9]. However, the phylogenetic inference of these results remained as the ambiguous species relationship in the third evolutionary lineage of *Allium*; more individuals and morphological data are needed to further explore phylogenetic relationships of this complex group.

### 4.2. Comparative Analysis of Eight Chloroplast Genomes

By analyzing genome size, GC contents, and gene numbers, high genome conservation was detected. In all chloroplast genomes, IR regions had the highest GC content; this may attribute to the presence of eight rRNA sequences in these regions (Table 1). The *rps16* gene and *rps2* gene were annotated as pseudogenes in *A. pallasii*, but they were annotated as protein-coding genes in the other seven cp genomes; it was reported that *rps16* was a pseudogene in *A. obliquum* and *A. sativum*, and the *rps2* was a pseudogene in more species [54]. These studies may be helpful to systematically understand the gene number, gene order, and chloroplast genome structure of *Allium* species.

The change in position of the IR/LSC and IR/SSC boundary may be caused by contraction or expansion of the IR region, and it is common in most of angiosperms [24, 55, 56]. The IR/SSC and IR/LSC boundary regions in these *Allium* species showed similar characteristics, excepting *A. pallasii*, which exhibited differences compared with other seven species. For example, the length from *psbA* gene to the LSC/IRb boundary in *A. pallasii* (627 bp) was much longer than that in other species (67–127 bp). Other genes, such as *rpl22*, *ycf1*, and *ndhF*, also had some differences in length and position. The differences in *A. pallasii* were consistent with our phylogenetic analysis; these seem to reflect far phylogenetic relationships between *A. pallasii* and other species in our study. The LSC-IR borders of eight *Allium* species were different from those of *Agapanthus coddii* and *Narcissus poeticus* by showing expansions, which may help in the prevention of gene loss-and-gain events [57]; these expansion or contraction of IR into LSC/SSC regions can often be observed in angiosperm plastomes [20, 57, 58]. The expansion and contraction of the IR region are related to the mutational hotspots; the highly divergent region *ycf1* is located in the SSC/IR boundary.

SSRs, thought to be the results of slipped strand mispairing during DNA replication, which are 16 bp repeating sequences in the chloroplast genome, have been always used as the molecular markers because of their high variability [18, 51, 59, 60]. The majority of these SSRs consisted of mono- and dinucleotide repeats, and most SSRs are located in the LSC region; the same results have appeared in previous research studies [28]. This uneven distribution of SSRs suggests that the difference of SSR numbers between *A. pallasii* and other species may be related to its short LSC region. For the wide use of SSRs in population studies, our new research could be used for studies on population genetics of this genus. Taking *A. cepa* as the reference, the mVISTA software was used to compare the complete chloroplast genome of these eight species. Seven highly divergent regions were selected for their greater nucleotide diversity, which might be more suitable sequences for developing potential molecular markers and species identification.

### 4.3. The Adaptation Evolution of *Allium* Species in Sect. *Pallasia* and Related Species

The pairwise Ka/Ks ratios are widely used as an effective way to detect positive selection or adaptive evolution in species, and the adaptation evolution of chloroplast genes to diverse ecological habitats of sunlight preferences has been reported in a recent study [27]. The Ka/Ks analysis of chloroplast genome genes in *Allium* species has also been reported, which suggested that *Allium* species may have undergone some selective forces in the evolutionary process [30].

Here, 42 photosynthesis genes of these 30 *Allium* species were concatenated, and higher pairwise Ka/Ks ratios were found in *A. schoenoprasoides* compared to *A. caeruleum* and *A. macrostemon* while a lower value of Ka/Ks ratios was detected between *A. caeruleum* and *A. macrostemon*, which may be caused by their different altitudes. *A. caeruleum* and *A. macrostemon* usually live in grass with an elevation of 500 m [5, 8], while *A. schoenoprasoides* lives in mountain pastures 3000 m above the sea level. A similar Ka/Ks ratio is also obtained between *A. tanguticum* (living in 3000 m) and *A. delicatulum* (living in 1500 m). The adaptation to different altitudes may lead to these higher pairwise Ka/Ks ratios. The Ka/Ks value difference among species is accompanied by great altitude difference; this seemed to reflect that the adaptive evolution of photosynthesis genes was mainly related to altitude or temperature, which has been identified in the previous study [61].

## 5. Conclusions

Here, we sequenced, assembled, and annotated eight chloroplast genomes of *Allium* with high-throughput sequencing technology. All eight cp genomes showed a typical quadripartite structure in length, gene content, gene order, and GC content similar among these sequences. Seven highly divergent regions were selected for their greater nucleotide diversity, which can be used to develop useful markers for future phylogenetic analysis. The maximum likelihood and BI phylogenetic results showed that *A. caeruleum*, *A. schoenoprasoides*, and *A. macrostemon* have close relationships with high support values.

Ka/Ks analysis indicated that the adaptation to different altitudes may lead to these higher pairwise Ka/Ks ratios. In conclusion, our results not only will be valuable to understanding the relationship between these eight *Allium* species but also provide useful cp genome resources for *Allium* phylogenetic study.

## Figures and Tables

**Table 1:** The length and GC contents of the whole genome sequences, SSC, LSC, and IR regions and the number of the genes.

Species	Length (bp)				GC contents (%)				Number of genes			
	Genome	SSC	LSC	IR	Genome	SSC	LSC	IR	Total	CDS	rRNA	tRNA
*A. caeruleum*	152967	18066	81884	26510	36.8	29.4	34.6	42.6	130	85	8	37
*A. delicatulum*	152984	17924	82046	26506	36.7	29.4	34.5	42.6	130	85	8	37
*A. macrostemon*	153158	17600	82700	26429	36.7	29.1	34.6	42.7	130	85	8	37
*A. pallasii*	151672	17665	80922	26541	36.6	29.1	34.4	42.5	128	83	8	37
*A. schoenoprasoides*	152729	18016	81678	26483	36.8	29.3	34.6	42.7	130	85	8	37
*A. songpanicum*	153247	18021	82537	26343	36.8	29.5	34.6	42.6	130	85	8	37
*A. tanguticum*	153024	17899	82123	26500	36.8	29.5	34.5	42.6	130	85	8	37
*A. teretifolium*	153340	17997	82281	26533	36.8	29.5	34.7	42.7	130	85	8	37

Abbreviations: CDS: protein-coding sequences/genes; LSC: large single-copy region; SSC: small single-copy region; IR: inverted repeat regions.

**Table 2:** Gene contents in eight *Allium* species.

Category	Group	Name
Self-replication	Large subunit of ribosome (LSU)	*rpl2(2)*, *rpl14*, *rpl16*, *rpl20*, *rpl22*, *rpl23(2)*, *rpl32*, *rpl33*, *rpl36*
	Small subunit of ribosome (SSU)	*rps2* ^∗^, *rps3*, *rps4*, *rps7(2)*, *rps8*, *rps11*, *rps12(2)*, *rps14*, *rps15*, *rps16*^∗^, *rps18*, *rps19(2)*
	DNA-dependent RNA polymerase	*rpoA*, *rpoB*, *rpoC1*, *rpoC2*
	Ribosomal RNA	*rrn4.5 (2)*, *rrna5(2)*, *rrn16(2)*, *rrn23(2)*
	Transfer RNAs (tRNA)	*trnA-UGC(2)*, *trnC-GCA*, *trnD-GUC*, *trnE-UUC*, *trnF-GAA*, *trnfM-CAU*, *trnG-GCC*, *trnG-UCC*, *trnH-GUG(2)*, *trnI-CAU(2)*, *trnI-GAU(2)*, *trnK-UUU*, *trnL-CAA(2)*, *trnL-UAA*, *trnL-UAG*, *trnM-CAU*, *trnN-GUU(2)*, *trnP-UGG*, *trnQ-UUG*, *trnR-ACG(2)*, *trnR-UCU*, *trnS-GCU*, *trnS-GGA*, *trnS-UGA*, *trnT-GGU*, *trnT-UGU*, *trnV-GAC(2)*, *trnV-UAC*, *trnW-CCA*, *trnY-GUA*
Photosynthesis	Photosystem I	*psaA*, *psaB*, *psaC*, *psaI*, *psaJ*
	Photosystem II	*psbA*, *psbB*, *psbC*, *psbD*, *psbE*, *psbF*, *psbH*, *psbI*, *psbJ*, *psbK*, *psbL*, *psbM*, *psbN*, *psbT*, *psbZ*
	Subunits of NADH-dehydrogenase	*ndhA*, *ndhB(2)*, *ndhC*, *ndhD*, *ndhE*, *ndhF*, *ndhG*, *ndhH*, *ndhI*, *ndhJ*, *ndhK*
	Subunits of cytochrome b/f complex	*petA*, *petB*, *petD*, *petG*, *petL*, *petN*
	Subunits of ATP synthase	*atpA*, *atpB*, *atpE*, *atpF*, *atpH*, *atpI*
	Large subunit of rubisco	*rbcL*
	Translation initiation factor	*infA*
Other genes	ATP-dependent protease subunit p gene	*clpP*
	Maturase	*matK*
	Envelope membrane protein	*cemA*
	Subunit of acetyl-CoA-carboxylase	*accD*
	C-type cytochrome synthesis gene	*ccsA*
	Hypothetical chloroplast reading frames (ycf)	*ycf1(2)*, *ycf2(2)*, *ycf3*, *ycf4*

Note: the gene names *rps2* and *rps16* with ^∗^ show the missing gene in *A. pallasii*.

## Data Availability

All data are openly available in Genebank, and the accession numbers have been showed in Supplementary Materials Table S1.

## Supplementary Materials

Supplementary materials Table S1: the accession number and collection locations of genomes in this study; genome of *Allium macrostemon* was sequenced in our previous study. Table S2: contig information generated by NOVOPlasty. Table S3: the accession number of the other species used in the phylogenetic analysis. Table S4: RSCU value in eight *Allium* chloroplast genome CDS. Figure S1: the phylogenetic relationships of these eight *Allium* species with other 22 related species based on combined coding sequences (A) and LSC regions (B). Tree constructed by Bayesian inference (BI) and maximum likelihood (ML) with the posterior probabilities of BI and the bootstrap values of ML above the branches, respectively. ∗ represent maximum support in all two analyses. Figure S2: pairwise Ka/Ks ratios of rbcL gene in *Allium* (Allioideae) and their outgroups (A). Pairwise Ka/Ks ratios of clpP gene in *Allium* (Allioideae) and their outgroups (B).
