# Supplementary material for: Adaptation Evolution and Phylogenetic Analyses of Species in Chinese Allium Section Pallasia and Related Species Based on Complete Chloroplast Genome Sequences
Source: Biomed Res Int. 2020 Jun 11;2020:8542797. doi: 10.1155/2020/8542797 (PMC7306069; doi:10.1155/2020/8542797)
Supplement: Supplementary materials — Table S1: the accession number and collection locations of genomes in this study; genome of Allium macrostemon was sequenced in our previous study. Table S2: contig information generated by NOVOPlasty. Table S3: the accession number of the other species used in the phylogenetic analysis. Table S4: RSCU value in eight Allium chloroplast genome CDS. Figure S1: the phylogenetic relationships of these eight Allium species with other 22 related species based on combined coding sequences (A) and LSC regions (B). Tree constructed by Bayesian inference (BI) and maximum likelihood (ML) with the posterior probabilities of BI and the bootstrap values of ML above the branches, respectively. ∗ represent maximum support in all two analyses. Figure S2: pairwise Ka/Ks ratios of rbcL gene in Allium (Allioideae) and their outgroups (A). Pairwise Ka/Ks ratios of clpP gene in Allium (Allioideae) and their outgroups (B). [file 8542797.f1.pdf]

Table S1. The accession number and collecting locations of genomes in this study, genome of *Allium macrostemon* was sequenced in our previous study.

Name	Accession	Locality	Altitude(m)	Voucher Specimen
Allium caeruleum	MN648630	Urumqi, Xinjiang	900	XFM20180607-1
Allium delicatulum	MN648631	Urumqi, Xinjiang	1700	XFM20180612-1
Allium macrostemon	MK751472	Mountain Baohua, Jiangsu	300	XFM20180817-2
Allium pallasii	MN648632	Urumqi, Xinjiang	900	XFM20180612-3
Allium schoenoprasoides	MN648633	Khorgos, Xinjiang	3000	XFM20180801-4
Allium songpanicum	MN648634	Songpan, Sichuan	2700	XFM20190806-1
Allium tanguticum	MN648635	Baiyu, Sichuan	3000	XFM20190820-1
Allium teretifolium	MN648636	Tekes, Xinjiang	1800	XFM20180726-1

Table S2. Contigs information generated by Novoplasty.

	A. caeruleum	*A. delicatulum*	*A. macrostemon*	*A. pallasii*	*A. schoenoprasoides*	*A. songpanicum*	*A. tanguticum*	*A. teretifolium*
Total reads	16331770	16331770	16331770	16331770	16331770	16331770	16331770	16331770
Aligned reads	338998	292058	240938	505350	348678	204560	164472	496734
Assembled reads	308872	272528	214352	469472	317174	184650	149372	471050
Organelle genome %	2.08%	1.79%	1.48%	3.09%	2.13%	1.25%	1.01%	3.04%
Average organelle coverage	335	288	238	503	345	202	162	489
Insert range	1.9	1.9	1.9	1.9	1.9	1.9	1.9	1.9
Insert range strict	1.3	1.3	1.3	1.3	1.3	1.3	1.3	1.3
Subsampled fraction %	47.44%	31.56%	35.39%	36.71%	44.44%	33.60%	38.93%	39.72%
Total contigs	3	3	3	3	3	3	3	3
Largest contig	134896 bp	135060 bp	134980 bp	133943 bp	134713 bp	135226 bp	135125 bp	135343 bp
Smallest contig	19090 bp	18917 bp	19112 bp	18691 bp	19021 bp	19021 bp	18892 bp	18991 bp
Average insert size	303 bp	313 bp	324 bp	346 bp	306 bp	316 bp	337 bp	358 bp

Table S3. The accession number of the other species using in the phylogenetic analysis.

Name	Accession	Name	Accession
Allium altaicum	NC_040972	*Allium oschaninii*	MK411816
Allium cepa	KM088014	*Allium paradoxum*	NC_039661
Allium chinense	MK096442	*Allium platyspathum*	LT673892
Allium chrysanthum	MH992108	*Allium prattii*	MG739457
Allium chrysocephalum	NC_042155	*Allium pskemense*	MK411815
Allium fistulosum	NC_040222	*Allium rude*	NC_042158
Allium herderianum	MH992110	*Allium tuberosum*	MK335929
Allium maowenense	MH992111	*Allium ursinum*	MH157875
Allium monanthum	MH748538	*Allium victorials*	NC_037240
Allium obliquum	MG670111	*Allium xichuanense*	NC_042159

Table S4 RSCU value in eight *Allium* chloroplast genomes CDS.

AA	codon	Aca	Ade	Ama	Apa	Ash	Aso	Ata	Ate
Phe	UUC	0.68	0.68	0.68	0.68	0.68	0.68	0.67	0.68
	UUU	1.32	1.32	1.32	1.32	1.32	1.32	1.33	1.32
Leu	CUG	0.43	0.45	0.43	0.42	0.42	0.43	0.44	0.43
	CUC	0.54	0.51	0.51	0.53	0.53	0.54	0.52	0.53
	CUA	0.69	0.64	0.67	0.67	0.69	0.68	0.68	0.68
	UUG	1.39	1.41	1.37	1.39	1.38	1.37	1.36	1.37
	CUU	1.39	1.45	1.43	1.41	1.42	1.41	1.41	1.43
	UUA	1.56	1.53	1.59	1.57	1.56	1.58	1.6	1.57
Ile	AUA	0.69	0.6	0.7	0.69	0.69	0.7	0.71	0.69
	AUC	0.72	0.78	0.73	0.72	0.72	0.71	0.71	0.71
	AUU	1.58	1.62	1.58	1.59	1.59	1.59	1.58	1.6
Met	AUG	1	1	1	1	1	1	1	1
Val	GUC	0.64	0.66	0.63	0.64	0.63	0.61	0.61	0.63
	GUG	0.65	0.66	0.63	0.65	0.65	0.65	0.64	0.66
	GUA	1.02	0.98	1	0.99	1	1.01	1.01	1.02
	GUU	1.7	1.71	1.73	1.72	1.72	1.73	1.73	1.69
Ser	UCG	0.78	0.77	0.76	0.78	0.78	0.78	0.76	0.77
	AGC	0.78	0.82	0.81	0.8	0.81	0.8	0.8	0.8
	UCC	0.9	0.92	0.9	0.9	0.9	0.9	0.9	0.9
	AGU	0.96	0.97	0.97	0.97	0.96	0.97	1	0.96
	UCU	1.14	1.1	1.13	1.12	1.13	1.14	1.11	1.13
	UCA	1.43	1.42	1.43	1.42	1.41	1.42	1.43	1.42
Pro	CCG	0.67	0.72	0.69	0.67	0.68	0.68	0.68	0.66
	CCU	1.19	1.13	1.14	1.17	1.14	1.13	1.2	1.21
	CCA	1.32	1.32	1.3	1.34	1.33	1.32	1.28	1.32
Thr	ACC	0.79	0.85	0.84	0.81	0.82	0.81	0.83	0.82
	ACG	0.79	0.81	0.78	0.78	0.78	0.78	0.77	0.8
	ACU	1.09	1.06	1.07	1.07	1.09	1.08	1.1	1.07
	ACA	1.33	1.28	1.31	1.34	1.31	1.32	1.3	1.31
Ala	GCG	0.6	0.59	0.58	0.62	0.59	0.58	0.6	0.6
	GCC	0.74	0.72	0.69	0.73	0.69	0.71	0.69	0.71
	GCU	1.24	1.28	1.29	1.28	1.3	1.28	1.3	1.28
	GCA	1.42	1.41	1.45	1.37	1.42	1.43	1.41	1.41
Tyr	UAC	0.58	0.6	0.58	0.58	0.58	0.58	0.57	0.58
	UAU	1.42	1.4	1.42	1.42	1.42	1.42	1.43	1.42
His	CAC	0.6	0.64	0.62	0.62	0.61	0.63	0.6	0.6
	CAU	1.4	1.36	1.38	1.38	1.39	1.37	1.4	1.4
Gly	GGC	0.72	0.76	0.74	0.74	0.73	0.75	0.71	0.73
	GGU	0.87	0.81	0.83	0.85	0.85	0.85	0.84	0.84
	GGG	0.99	1.05	1.02	1	0.99	1	1.02	1.03
	GGA	1.42	1.38	1.41	1.4	1.43	1.4	1.43	1.4

Gln	CAG	0.55	0.59	0.56	0.54	0.54	0.53	0.52	0.55
	CAA	1.45	1.41	1.44	1.46	1.46	1.47	1.48	1.45
Asn	AAC	0.57	0.58	0.56	0.57	0.57	0.56	0.58	0.57
	AAU	1.43	1.42	1.44	1.43	1.43	1.44	1.42	1.43
Lys	AAG	0.56	0.61	0.56	0.57	0.57	0.56	0.56	0.57
	AAA	1.44	1.39	1.44	1.43	1.43	1.44	1.44	1.43
Asp	GAC	0.46	0.49	0.47	0.48	0.47	0.47	0.47	0.49
	GAU	1.54	1.51	1.53	1.52	1.53	1.53	1.53	1.51
Glu	GAG	0.61	0.65	0.61	0.59	0.6	0.6	0.58	0.61
	GAA	1.39	1.35	1.39	1.41	1.4	1.4	1.42	1.39
Cys	UGC	0.91	0.92	0.9	0.91	0.91	0.9	0.91	0.91
	UGU	1.09	1.08	1.1	1.09	1.09	1.1	1.09	1.09
Trp	UGG	1	1	1	1	1	1	1	1
Arg	CGC	0.41	0.41	0.4	0.4	0.39	0.38	0.39	0.42
	CGU	0.55	0.55	0.58	0.57	0.57	0.58	0.55	0.57
	CGG	0.6	0.65	0.62	0.62	0.63	0.63	0.6	0.64
	CGA	0.84	0.8	0.8	0.82	0.82	0.81	0.83	0.82
	AGG	1.38	1.44	1.38	1.41	1.4	1.39	1.41	1.37
	AGA	2.22	2.15	2.23	2.18	2.19	2.21	2.22	2.18
TER	UAG	0.73	0.73	0.71	0.73	0.73	0.72	0.74	0.72
	UAA	1.01	1.02	1.03	1.01	1.02	1.03	1.01	1.03
	UGA	1.25	1.24	1.26	1.26	1.25	1.25	1.25	1.26

RSCU represents relative synonymous codon usage. Aca, Ade, Ama, Apa, Ash, Aso, Ata and Ate stand for *A. caeruleum*, *A. delicatulum*, *A. macrostemon*, *A. pallasii*, *A. schoenoprasoides*, *A. songpanicum*, *A. tanguticum* and *A. teretifolium*.

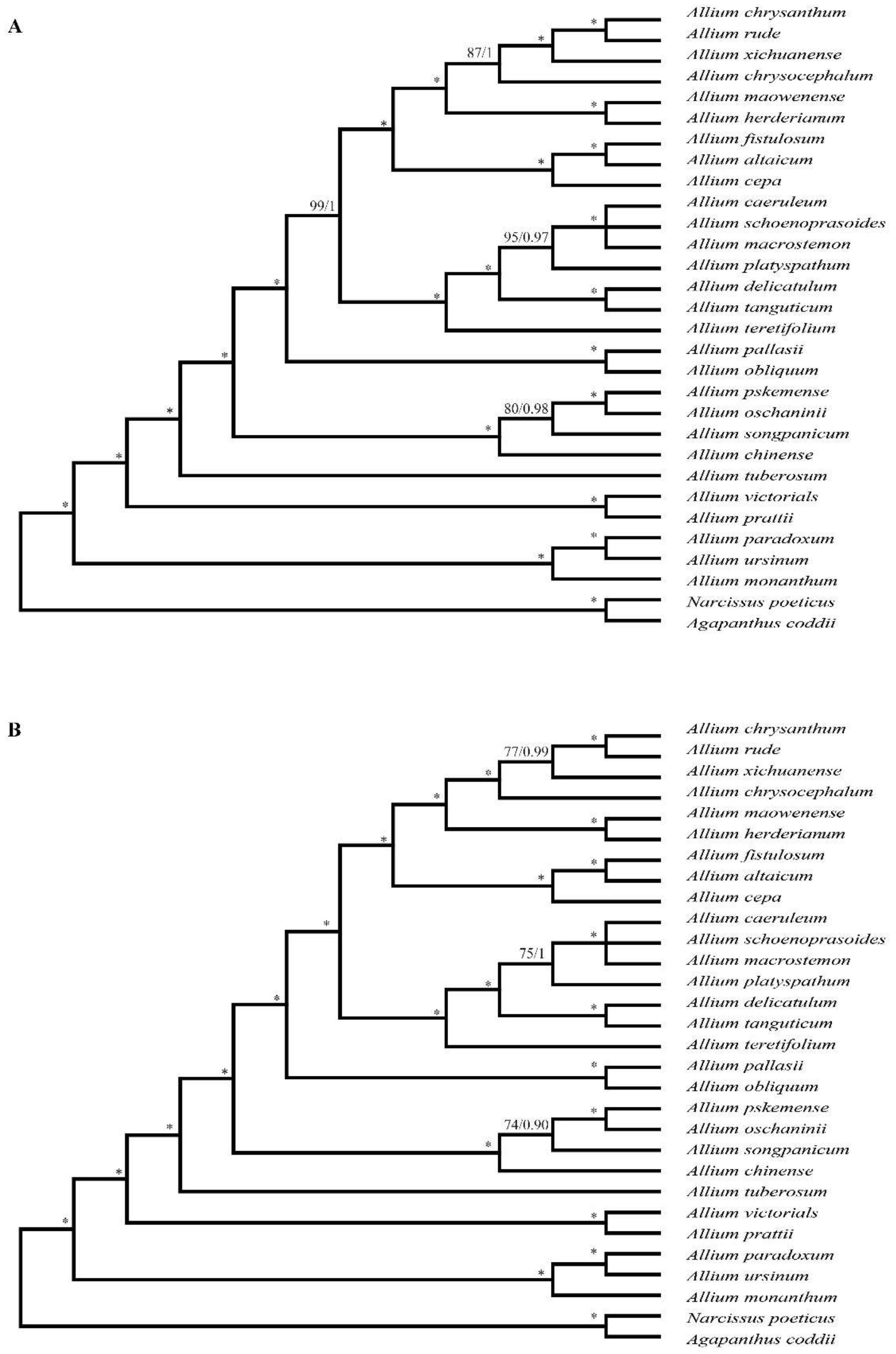

Figure S 1. The phylogenetic relationships of these eight *Allium* species with other 22 related species based on combined coding sequences (A) and LSC regions (B). Tree constructed by Bayesian inference (BI) and maximum likelihood (ML) with the posterior probabilities of BI and the bootstrap values of ML above the branches, respectively. * Represent maximum support in all two analyses.

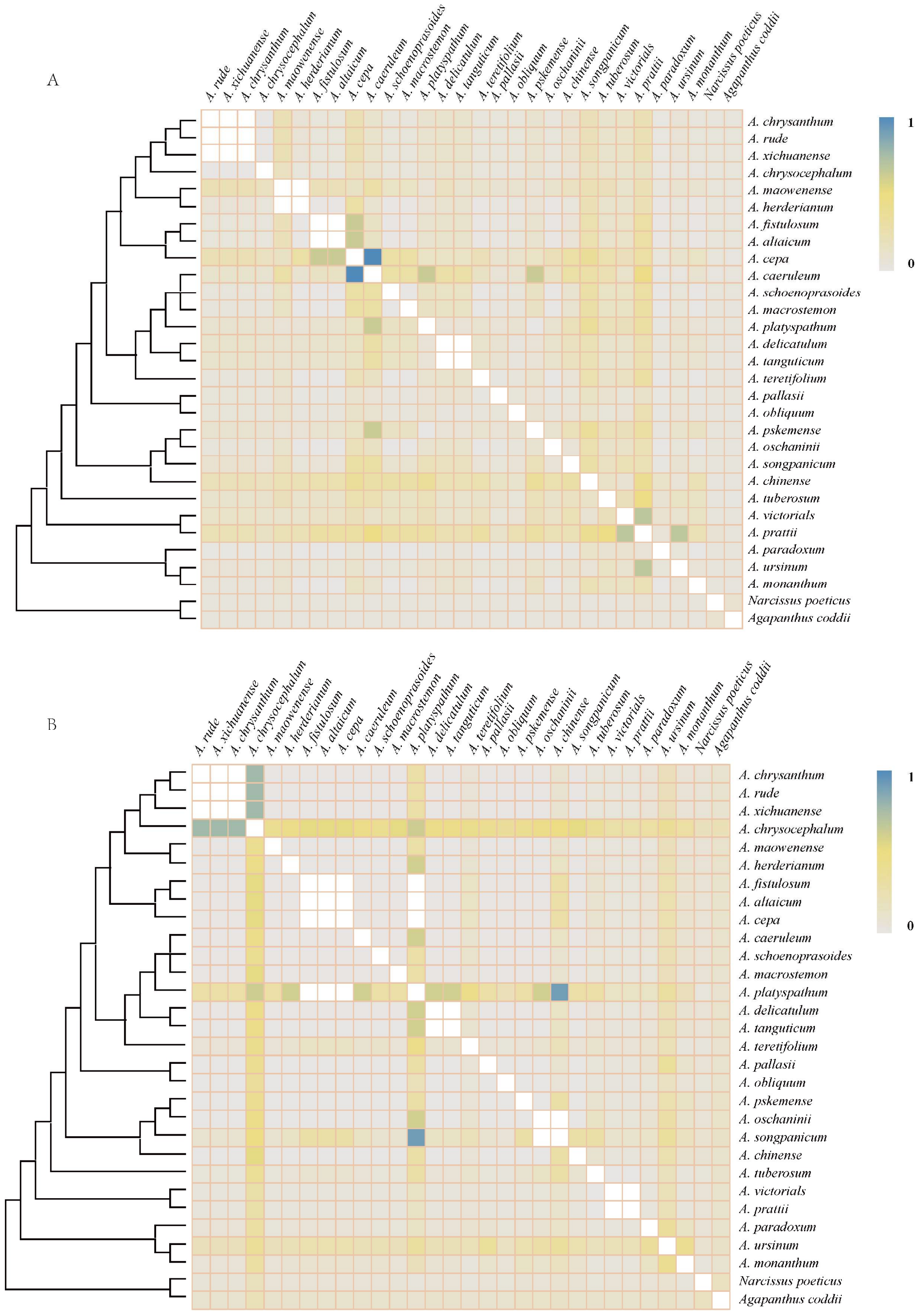

Figure S 2 Pairwise Ka/Ks ratios of *rbcL* gene in *Allium* (Allioideae) and their outgroups (A). Pairwise Ka/Ks ratios of *clpP* gene in *Allium* (Allioideae) and their outgroups (B).
